# Physicochemical data on aqueous polymeric systems of methyl cellulose and lambda- and kappa-carrageenan: SAXS, rheological, densitometry, and sound velocity measurements

**DOI:** 10.1016/j.dib.2017.09.025

**Published:** 2017-09-20

**Authors:** Jure Cerar, Iztok Dogsa, Andrej Jamnik, Matija Tomšič

**Affiliations:** a Faculty of Chemistry and Chemical Technology, University of Ljubljana, Večna pot 113, SI-1000 Ljubljana, Slovenia; b Biotechnical Faculty, University of Ljubljana, Večna pot 111, SI-1000 Ljubljana, Slovenia

**Keywords:** Food-grade polymers, Basic polymer characterization, Methyl cellulose, kappa-Carrageenan, lambda-Carrageenan

## Abstract

General as well as more specific physicochemical data obtained by studying the structure and various dynamical properties of aqueous polymer systems of methyl cellulose, λ−carrageenan, and κ−carrageenan are presented in graphical and numeric tabular form. The data provide basic polymer characterization info as also a specific structural and dynamical info for aqueous solutions of three industrially very important polymers (food additives) that are available commercially. The commercial availability has much bigger impact to applications, research and connected advances, when the basic substances are well characterized – a feature that is still missing for many commercially available polymers unfortunately.

**Specifications Table**

**Table:** 

Subject area	*Chemistry*
More specific subject area	*Physical Chemistry*
Type of data	*Figures, Data files*
How data was acquired	*Anton Paar density and sound velocity meter DSA 5000 (Prototype 5); Anton Paar Physica rheometer UDS 200, Anton Paar Physica MCR 302; In-lab modified Anton Paar “Old-Kratky” SAXS camera.*
Data format	*Raw and/or reduced data*
Experimental factors	*The polymer samples were used as purchased. They were dispersed in water by vigorous stirring at approximately 70 °C and then left to cool-down and rest overnight in the fridge at 4 °C.*
Experimental features	*Temperature dependencies of the measured properties were obtained in a step-wise manner, where the samples were left to equilibrate at a specific temperature for a certain time before an individual measurement.*
Data source location	*Ljubljana, Slovenia, Europe.*
Data accessibility	*Data is with this article. The numerical raw data files are provided in the Data in Brief DataVerse*, 10.7910/DVN/ZOE59W.

**Value of the data**

(1) These data represent a comprehensive data set on basic physicochemical and structural characterization of three industrially important polysaccharides that are commercially available. They should be of interest to the researchers using these polymers in their investigations to avoid the need of repetition of basic polymer characterization.
(2) The temperature dependent dynamic viscosity data reveal the transition temperatures and viscosity-change information of the gelling/de-gelling transitions that allow easier selection of the temperature regimes for further investigation and/or possible application of these and related systems.
(3) These specific SAXS data have already been evaluated by the classic and the string-of-beads model and, as here presented in a numerical tabular form they enable an interested researcher to use them for benchmarking of performance of other developed evaluation models or models in development.

## 1 Data

The data shown in this article are related to our recent structural study of aqueous polymer systems of methyl cellulose (MC), λ−carrageenan (LC), and κ−carrageenan (KC) utilizing the small-angle scattering (SAXS) study by the string-of-beads model [1] and on a general level in part also to some of our previous studies [2], [3], [4], [5], [6], [7], [8]. Some of them represent the basic polymer characterization and the others contain additional information on practically very important dynamic and structural properties of these systems.

**Fig. 1:**
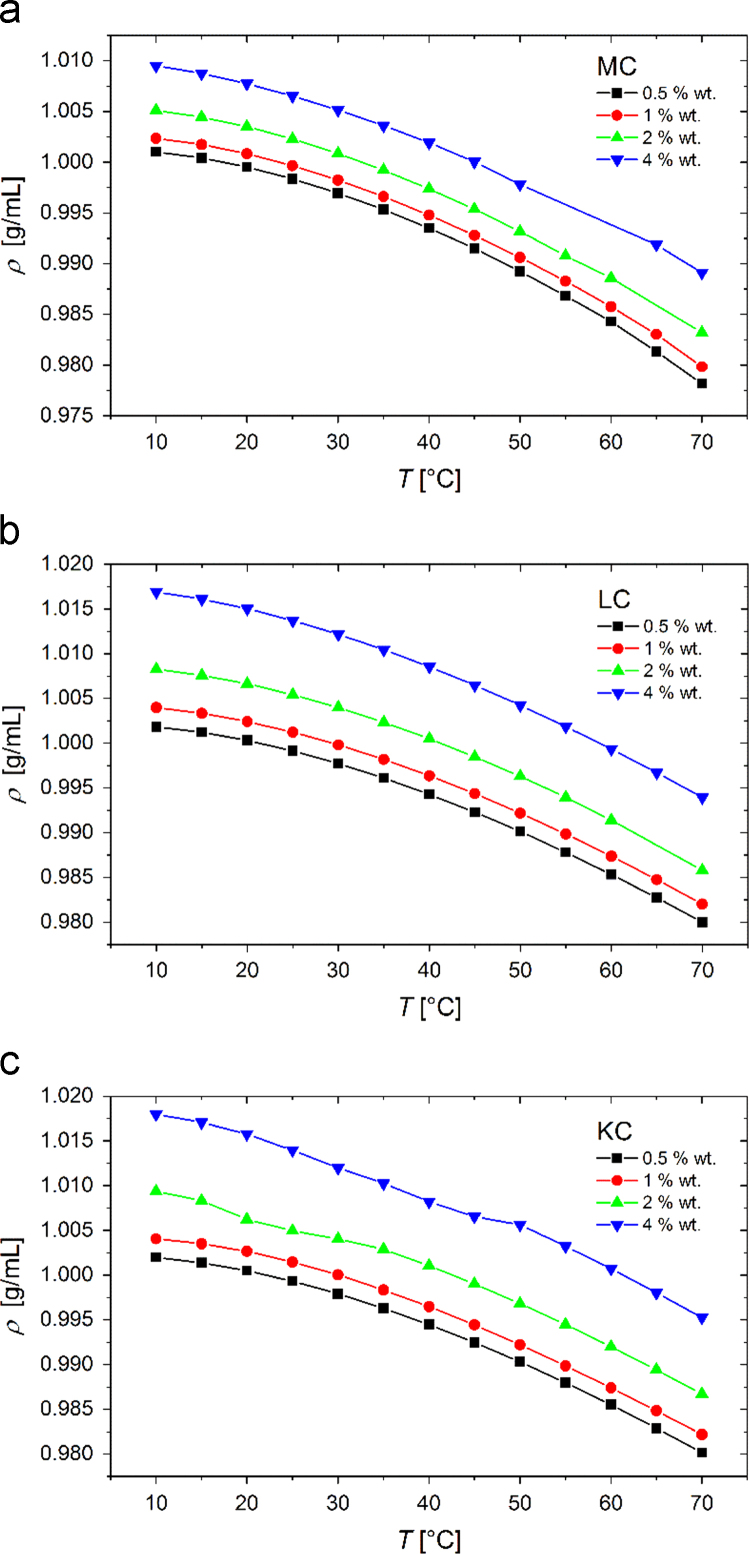
Experimental data for temperature dependence of the density of 0.5, 1, 2, and 4 wt% (a) MC, (b) LC, and (c) KC aqueous solutions.

**Fig. 2:**
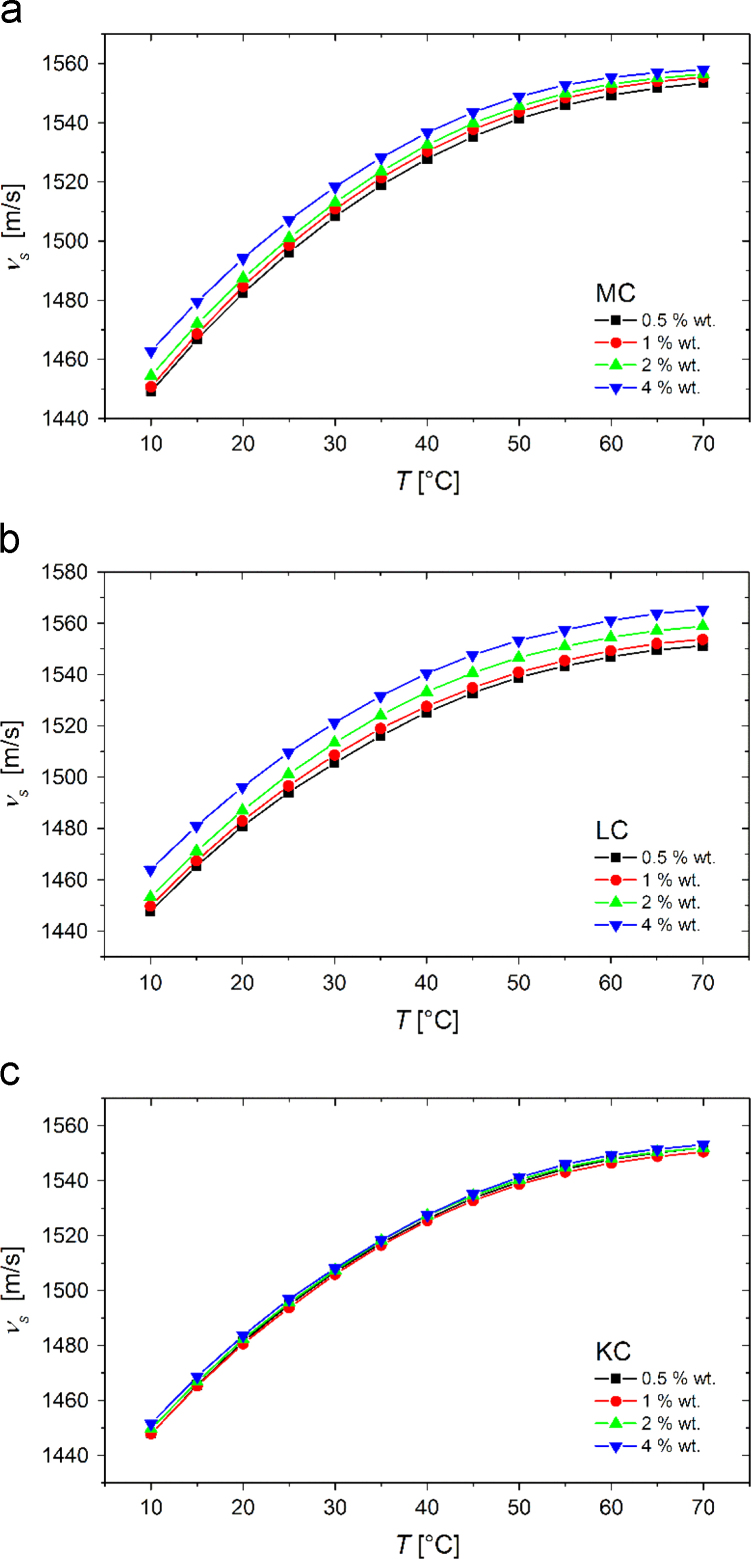
Experimental data for temperature dependence of sound velocity of 0.5, 1, 2,and 4 wt% (a) MC, (b) LC, and (c) KC aqueous solutions.

**Fig. 3:**
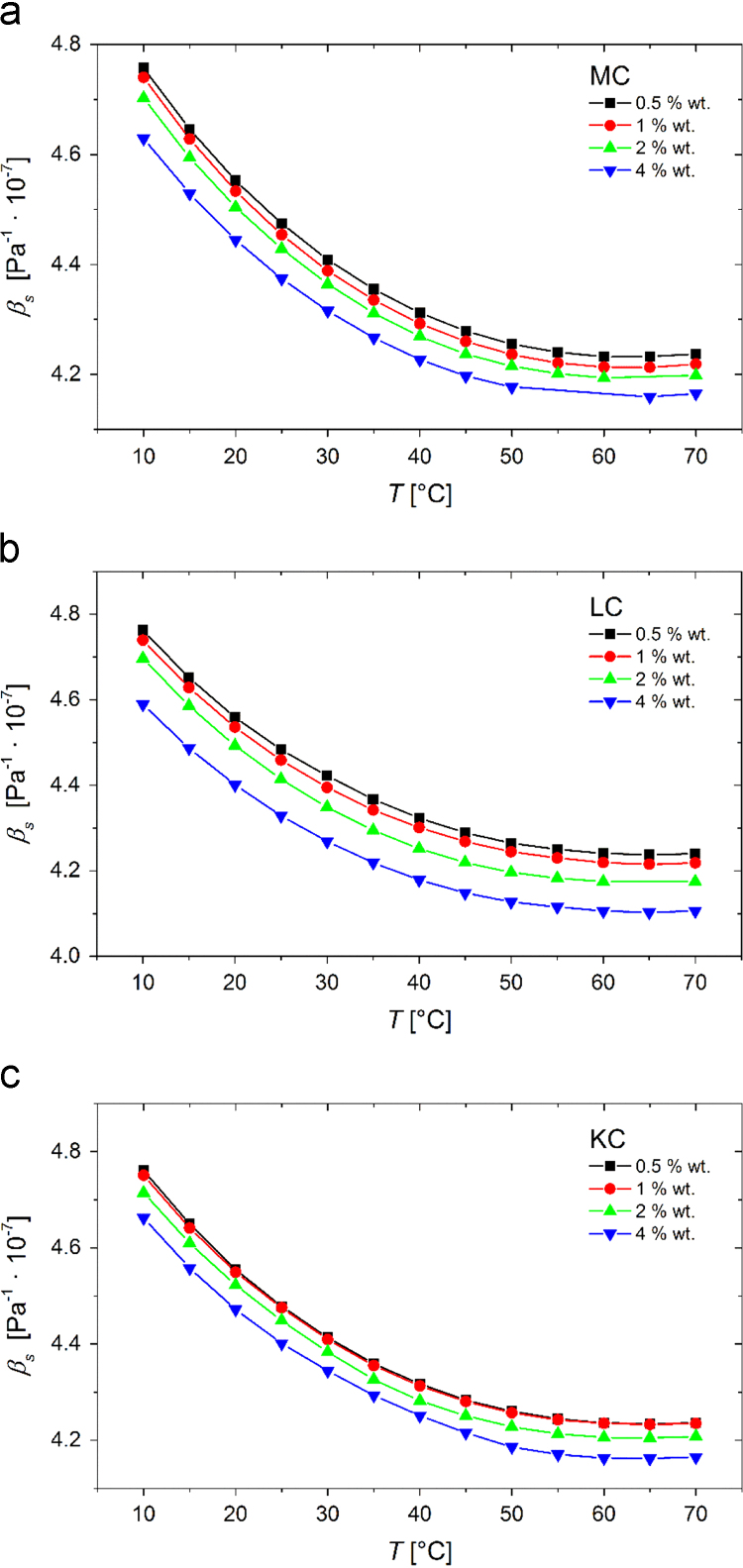
Temperature dependence of adiabatic compressibility of 0.5, 1, 2,and 4 wt% (a) MC, (b) LC, and (c) KC aqueous solutions.

In Fig. 1 we show the experimental temperature dependent density values, ρ, of 0.5, 1, 2, and 4 wt% MC, LC, and KC aqueous solutions and in Fig. 2 the corresponding experimental sound velocity data, $v_{s}$. According to the following expression:

(1) $$\beta_{S}=\frac{1}{\rho \cdot v_{s}^{2}}$$

data from Fig. 1, Fig. 2 can be reduced to yield the adiabatic compressibility values, $\beta_{S}$, which are plotted in Fig. 3. Data shown in Fig. 1, Fig. 2, Fig. 3 are available in the numerical tabular form in Supporting information (file: “Densitometry_data.xlsx”).

At this point, we need to comment the density data shown in Fig. 1 that were obtained at 60 °C for 2 wt% and in the temperature regime from 55 to 60 °C for 4 wt% MC sample, and from 20 to 30 °C for 2 wt% and from 40 to 50 °C for 4 wt% KC sample. For these samples in these specific temperature regimes, some minor difficulties with the density measurements were observed and were ascribed to the transition of the sol to the solid gel state. Interestingly, slight change in the density data trend can be observed in these regimes. These issues were not observed in the data for lower polymer concentrations, where the system form less firm gels.

**Fig. 4:**
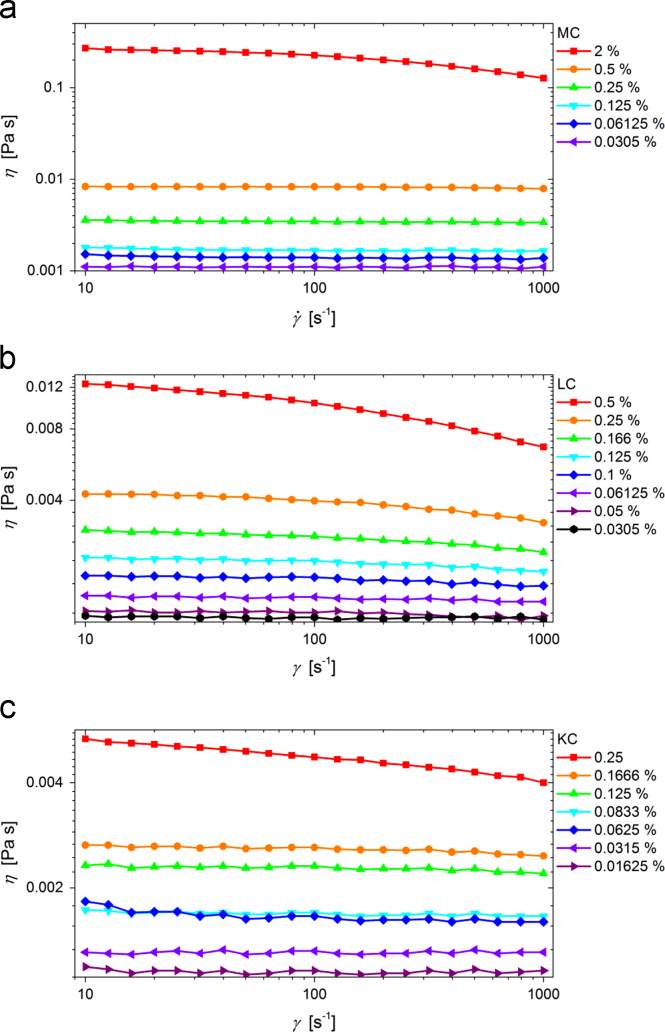
Experimental data on dynamic viscosity vs. shear rate of various concentrations of (a) MC, (b) LC, and (c) KC aqueous solutions.

**Fig. 5:**
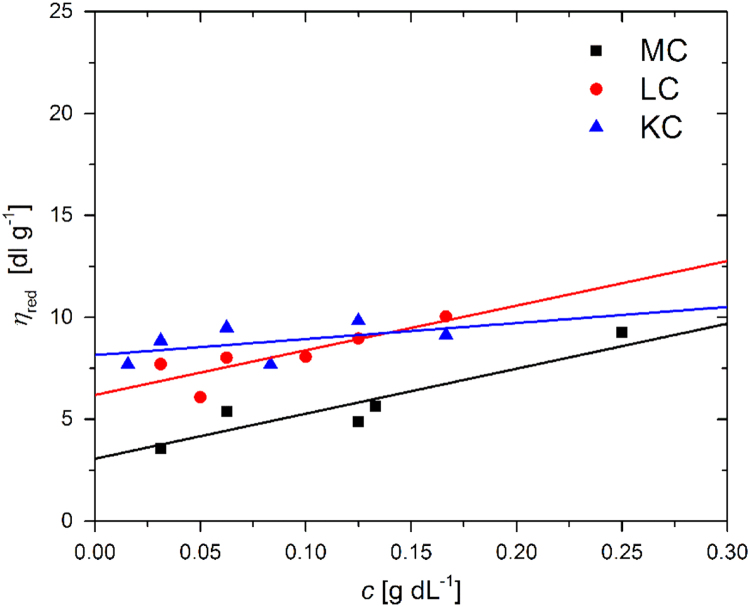
Determination of intrinsic viscosity: experimental data on reduced viscosity vs. polymer concentration for MC (black symbols), LC (red symbols), and KC (blue symbols).

Dynamic properties of the studied samples of MC, LC, and KC are revealed through the rheological results that are depicted in Fig. 4 in a form of dynamic viscosity, η, vs. shear rate, γ˙. These KC and LC samples were prepared in 0.1 M NaCl, while the MC samples are prepared in pure water. With the reduction of the data presented in Fig. 4 the reduced viscosity values, $\eta_{\mathrm{red}}$, are obtained according to the expression:

(2) $$\eta_{\mathrm{red}}=\frac{\eta -\eta_{o}}{\eta_{o}\cdot c},$$

where $\eta_{o}$ represents the viscosity of the solvent and c the polymer mass concentration, and are plotted in Fig. 5 as concentration dependence of the reduced viscosity. Based on the extrapolation to zero concentration the intrinsic viscosities, [η], are obtained and can be used to determine the average molecular mass of the polymers. Data depicted in Fig. 4, Fig. 5 are available in the numerical tabular form in Supporting information (file: “Rheological_data.xlsx”).

**Fig. 6:**
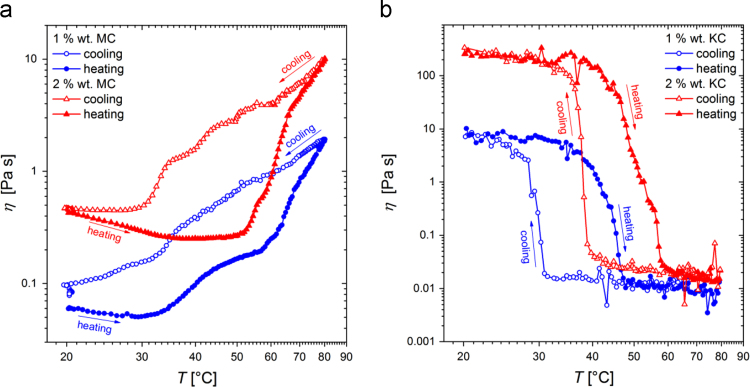
Experimental data for temperature dependence of dynamic viscosity of 1 and 2 wt% (a) MC and (b) KC aqueous solutions. Data were obtained at constant angular frequency of 3 rad s^−1^ and low shear strain (below 10% for MC and below 3% for KC samples).

Similarly, information on thermal influence on the viscosity of MC and KC aqueous solutions and the sol–gel and gel–sol transition temperatures is revealed through the dynamic viscosity data depicted in Fig. 6 and are available in the numerical tabular form in Supporting information (file: “Oscillatory_Rheological_data.xlsx”). In the latter file, also temperature dependence on the complex viscosity and its imaginary component are given. However, the corresponding additional oscillatory rheological and DSC data one can find in Ref. [2].

**Fig. 7:**
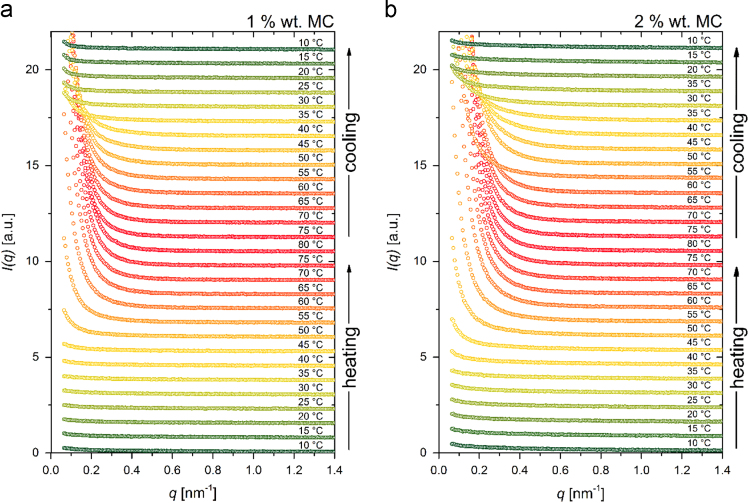
Experimental SAXS data of (a) 1 and (b) 2 wt% MC aqueous solutions obtained at different temperatures during the stepwise heating/cooling temperature cycle. For the sake of clarity, the data are shifted upwards by different constant factors.

**Fig. 8:**
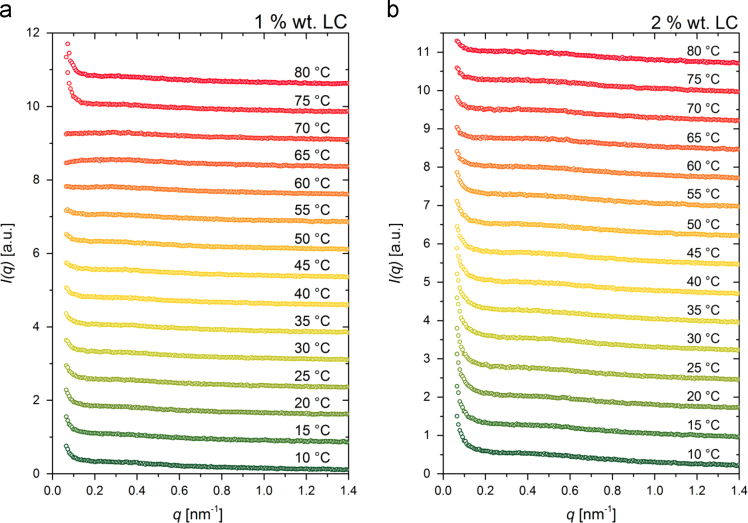
Experimental SAXS data of (a) 1 and (b) 2 wt% LC aqueous solutions obtained at different temperatures during the stepwise heating/cooling temperature cycle. For the sake of clarity, the data are shifted upwards by different constant factors.

**Fig. 9:**
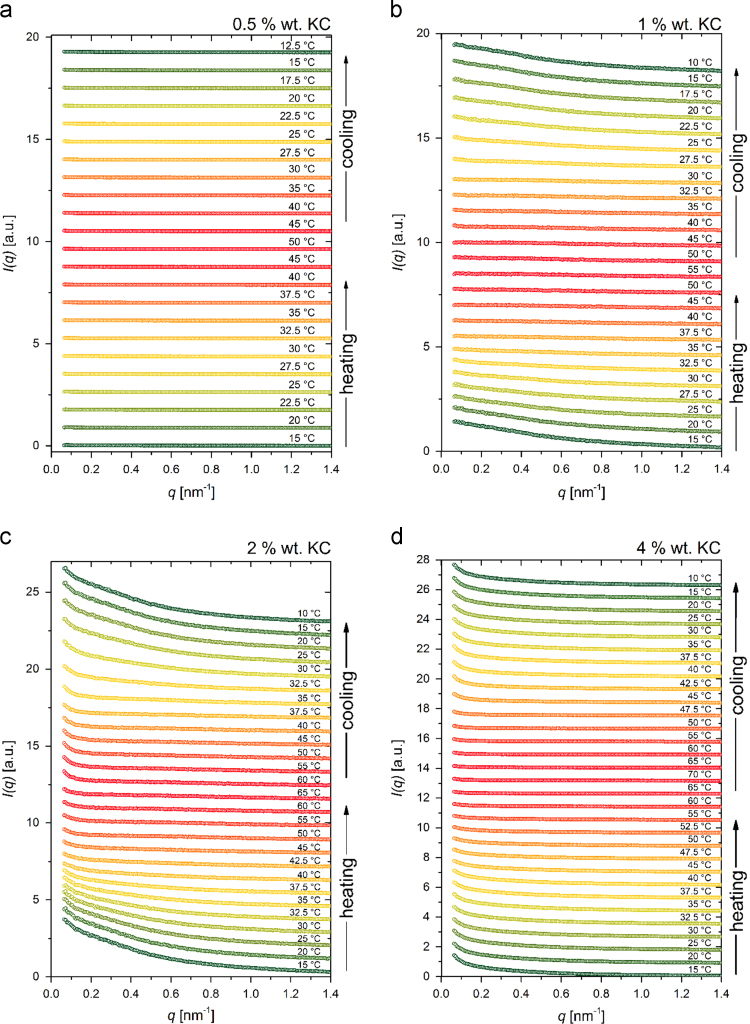
Experimental SAXS data of (a) 0.5, (b) 1, (c) 2, and (d) 4 wt% KC aqueous solutions obtained at different temperatures during the stepwise heating/cooling temperature cycle. For the sake of clarity, the data are shifted upwards by different constant factors.

In Fig. 7, Fig. 8, Fig. 9 we show the experimental SAXS data at different temperatures. They carry the information on the thermally induced structural changes of the MC, LC, and KC aqueous systems. The corresponding SAXS data put to an absolute scale using water as a secondary standard [9] are available in the Supporting information in a numerical tabular form (file: “SAXS_data.xlsx”), where also the length profile of the line-collimated primary beam is provided. Namely, even though scaled to an absolute scale, these data are still experimentally smeared due to the line-collimated primary beam. These SAXS data can be analyzed following different approaches. Some of the well-accepted ones have already been applied to some of these data and successfully yielded various more or less detailed information on the structure and dynamics in these systems [1], [3], the others are still under the development or will be developed in the future. The latter will certainly need a good direct comparative data for benchmarking of their performance and in this way facilitating their general acceptance.

## 2 Experimental design, materials and methods

### 2.1 Materials

The λ−carrageenan (Sigma Aldrich, SKU 22049), κ−carrageenan (Fluka BioChemica – Sigma Aldrich, SKU 22048), and methyl cellulose (food-grade Methocel A4C FG; Dow Chemical Company) were used as purchased, without further purification. The weight average molecular masses, $M_{w}$, of original polymer samples were determined utilizing the results for intrinsic viscosities of (6.2±0.6) dL g^−^^1^, (8.1±0.7) dL g^−^^1^, and (3.0±0.5) dL g^−^^1^ that were obtained by extrapolations as depicted in Fig. 5. These intrinsic viscosity values were used according to the Mark-Houwink equation:

(3) $$\left[\eta \right]=K\cdot M_{w}^{\alpha },$$

where α and K are the coefficients obtained from the literature [10], [11], [12], and yielded the weight average molecular masses of (320±60) kDa, (590±60) kDa, and (105±25) kDa for LC, KC, and MC, respectively. For this purpose we have used the following values for α and K: 0.6 and 3.07·10^−3^ for LC, values of 0.86 and 8.80·10^−5^ for KC, and values of 0.75 and 5.20·10^−^^4^ for MC, respectively. Since the intrinsic viscosities were in units of dL g^-1^ the weight average molecular masses were obtained in units of Da [10], [11], [12].

### 2.2 Sample preparation

First the aqueous stock solutions of LC, KC, and MC with 4 wt% of polymer were prepared by weighing, immediately heating to approximately 70 °C and vigorously stirring until the entire amount of polymer in the sample was successfully dispersed (to prevent lumping and allowing for all polymer particles to be properly wetted). Afterwards, the solutions were left to cool and were stored in a fridge at 4 °C overnight, which ensured enough time for the polymer to fully dissolve. The final samples for the SAXS measurements were prepared by diluting the stock solution with water – the KC stock solution was reheated to approximately 70 °C to melt the gel before such dilution. Deionized Milipore-quality water was used for the sample preparations. We have followed this procedure, because in a number of our previous studies it ensured well dissolved polymer samples and reproducible results on various studied properties of these and related systems [2], [4], [5], [6], [7], [8].

To determine the intrinsic viscosity values, the KC and LC samples were prepared in 0.1 M NaCl with polymer concentration ranging from 0.25% to 0.016% (w/v) [10], [11], [12]. Similarly, MC samples were prepared in pure water with polymer concentration ranging from 0.5% to 0.031% (w/v) [13].

### 2.3 Density and sound velocity measurements

The density and sound velocity measurements of sample solutions were performed utilizing the DSA 5000 instrument (prototype 5 instrument; Anton Paar, Graz) equipped with a density and sound velocity measuring cell. The measurements were performed in a stepwise temperature scan mode in heating direction. When at an individual step the set temperature was reached, a 2 min waiting equilibration time was used prior to 5 min long measurement.

### 2.4 Viscometry

Dynamic viscosity vs. shear rate data were measured on an Anton Paar Physica MCR 302 rotational cone-plate system with a plate diameter of 50 mm and are reported in a related Data in brief article. The distance between plates was 0.102 mm and the measuring temperature was (20.00 ± 0.05) °C. Approximately 590 µL of sample was applied to fill the gap between the plates. Flow curves at shear rates ranging from 10 to 1000 s^−1^ were measured. For calculating reduced viscosities the average viscosity in the shear range from 100 to 300 s^−1^ in the Newtonian flow regime was considered.

The temperature dependent dynamic viscosity values were measured on an Anton Paar Physica rheometer UDS 200, with a cone-plate geometry of 50 mm diameter and 2° cone angle during a temperature cycle between 20 and 80 °C with the scan speed of approximately 1 °C/min. All measurements were carried out in the oscillatory shear mode at an angular frequency of 3 rad/s and low shear strains to ensure the linearity of viscoelasticity. Prior to the data collection these settings were checked at different temperatures with the strain-sweep and angular frequency- sweep measurements. To reduce the water evaporation from the samples during the measurement a thin layer of low-viscosity silicone oil was applied on the peripheral surface.

### 2.5 Small-angle X-ray scattering measurements

Small-angle X-ray scattering spectra were measured in a modified Kratky camera (Anton Paar, Graz, Austria) equipped with a Göbel mirror, to obtain a focused, monochromatic beam, and a Mythen 1 K detector (Dectris, Baden, Switzerland) at a sample-to-detector distance of 310 mm. The X-ray generator (Philips, PW 3830/00) with a sealed X-ray tube (Cu $K_{\alpha }$; wavelength of 0.154 nm) was operated at 30 kV and 50 mA. The samples were placed in a standard quartz capillary with an outer diameter of 1 mm and a wall thickness of 10 μm. During the heating and cooling cycles the temperature was changed in a step-wise manner with steps of 2.5 or 5 °C. During each step the samples were thermostated (±0.1 °C) for 15 min using a Peltier element and then measured for an hour. In order to study the hysteresis of the sol-gel transformation, the temperature was changed from low to high temperatures and back. The scattering curves were normalized to the intensity of the primary beam and corrected for the scattering of the solvent and the background. The curves were further put to an absolute scale using water as a secondary standard – the resulting SAXS curves were, however, still experimentally smeared [9]. The obtained data is presented as a scattering function I(q), where q is the scattering vector q=4π/λ⋅sin(ϑ/2), with λ being the wavelength of the X-rays and ϑ the scattering angle. The experimental SAXS measurements were obtained in the following range of scattering vectors: 0.06 nm^−1^ < ϑ < 5 nm^−^^1^.
